# Automatic Seizure Detection and Prediction Based on Brain Connectivity Features and a CNNs Meet Transformers Classifier

**DOI:** 10.3390/brainsci13050820

**Published:** 2023-05-18

**Authors:** Ziwei Tian, Bingliang Hu, Yang Si, Quan Wang

**Affiliations:** 1 Key Laboratory of Spectral Imaging Technology, Xi’an Institute of Optics and Precision Mechanics, Chinese Academy of Sciences, Xi’an 710119, China; 2 School of Optoelectronics, University of Chinese Academy of Sciences, Beijing 101408, China; 3 Key Laboratory of Biomedical Spectroscopy of Xi’an, Xi’an Institute of Optics and Precision Mechanics, Chinese Academy of Sciences, Xi’an 710119, China; 4 Department of Neurology, Sichuan Academy of Medical Science and Sichuan Provincial People’s Hospital, Chengdu 610072, China; 5 School of Medicine, University of Electronic Science and Technology of China, Chengdu 611731, China

**Keywords:** epileptic state classification, EEG, brain connectivity, support vector machine, CNNs meet transformers

## Abstract

(1) Background: Epilepsy is a neurological disorder that causes repeated seizures. Since electroencephalogram (EEG) patterns differ in different states (inter-ictal, pre-ictal, and ictal), a seizure can be detected and predicted by extracting various features. However, the brain connectivity network, a two-dimensional feature, is rarely studied. We aim to investigate its effectiveness for seizure detection and prediction. (2) Methods: Two time-window lengths, five frequency bands, and five connectivity measures were used to extract image-like features, which were fed into a support vector machine for the subject-specific model (SSM) and a convolutional neural networks meet transformers (CMT) classifier for the subject-independent model (SIM) and cross-subject model (CSM). Finally, feature selection and efficiency analyses were conducted. (3) Results: The classification results on the CHB-MIT dataset showed that a long window indicated better performance. The best detection accuracies of SSM, SIM, and CSM were 100.00, 99.98, and 99.27%, respectively. The highest prediction accuracies were 99.72, 99.38, and 86.17%, respectively. In addition, Pearson Correlation Coefficient and Phase Lock Value connectivity in the β and γ bands showed good performance and high efficiency. (4) Conclusions: The proposed brain connectivity features showed good reliability and practical value for automatic seizure detection and prediction, which expects to develop portable real-time monitoring equipment.

## 1. Introduction

Epilepsy is a common neurological disorder worldwide, causing a huge burden to patients and their families. It is a transient brain dysfunction caused by sudden abnormal and super-synchronous discharges of neurons [1]. The neuronal discharge pattern of epilepsy generally goes through three stages: the inter-ictal, pre-ictal, and ictal phases [2]. Seizure detection refers to identifying the ictal phase, which is time-consuming for clinicians as it involves visually examining electroencephalogram (EEG) changes. A seizure detection model can improve detection efficiency and accuracy. Approximately 30% of the patients have intractable epilepsy [3]. The unpredictability of seizure recurrence leads to a serious psychosomatic impact. Therefore, a seizure prediction model is required to identify the pre-ictal phase to detect impending seizures [4]. The seizure prediction horizon (SPH) represents the time range within which seizures can be predicted in advance. The strategies of seizure detection, prediction model training, and validation fall into three categories: subject-specific model (SSM), subject-independent model (SIM), and cross-subject model (CSM).

There are two types of methods for detection and prediction. One is to feed raw EEG data into a neural network that automatically extracts features for classification [5,6]. This method requires large training samples, and the black-box nature of neural networks renders the learned features elusive. Therefore, we focused on the second method, which classifies manually designed features using machine or deep learning. The key to success is the effectiveness of the features, which are later divided into non-image and image features. Non-image features refer to one-dimensional features such as amplitude and spectrum power [7]. Short-time Fourier Transform (STFT) is the most popular method for transforming one-dimensional time series into two-dimensional images that can be successfully fed into a convolutional neural network (CNN) for large-sample image classification. Truong et al. [8] adopted a CNN as a classifier and time-frequency images as features to predict seizures, reaching a sensitivity of 81.20% and specificity of 84%. Zhang et al. [9] used a residual network (ResNet) to build SIM for seizure detection, with an accuracy of 96.71%. However, STFT is not applicable to resting-state EEG because its calculation cost is high for high-density EEGs [10].

In 2002, Spencer systematically described epilepsy as a brain network disease with functional and structural connectivity in the cortical and subcortical areas [11]. Hence, we speculated that the brain network composed of the connectivity [12,13] between each pair of brain regions could be another feature candidate. Some scientists have recently applied brain network features for seizure detection and prediction. Akbarian et al. [14] calculated three weight networks and extracted ten graph theory-based features after network binarization, yielding a detection accuracy of 99.43%. Zhang et al. [15] applied a similar method to predict seizures and achieved an accuracy of 89.2%. However, the studies above had three deficiencies. First, network binarization involves the experience-based setting of the threshold, which directly affects the effectiveness of features. Second, such non-image-like features cannot take advantage of CNNs. Third, additional steps such as binarization and graph theory feature extraction increase the complexity and time cost. Therefore, a few recent studies have begun to input brain networks as image-like features. For example, Zhang et al. [16] attempted to feed a weight brain network into a CNN for subject-specific prediction, achieving an accuracy of 89.98%. However, they did not build SIM and CSM to conduct a comprehensive evaluation, nor did they explore the effectiveness of other popular connectivity measures. 

The number of rows and columns of a brain connection matrix is determined by the number of electrodes, usually more than nineteen. Each element expresses information about an edge between two nodes. A complete matrix can represent an entire brain network, which includes some subnetworks involving many nodes and edges. This characteristic requires a larger receptive field to capture the long-range relationship between edges. However, traditional CNNs usually have limited receptive fields, which do not apply to such brain connectivity matrices, although they are image-like features. On the contrary, transformers commonly used in natural language processing often have a larger receptive field and can simultaneously process all the positional information of the input tensor [17]. Therefore, transformers attract increasing attention in computer vision [18,19,20]. Nevertheless, the self-attention modules in transformers require higher computational and memory costs, which cannot satisfy the efficiency requirement for the clinical application. The CNNs meet transformers (CMT) model proposed by Guo et al. [21] in 2022 could mitigate this problem by combing the advantages of both CNN and transformer. Experiments on ImageNet benchmarks and downstream tasks shows that use of CMT improves performance and reduces computational overhead.

Here, we propose, select, and evaluate brain connectivity features for seizure detection and prediction. Considering sample sizes under different training strategies and the efficiency in practical applications, we adopt SVM as the classifier for SSM and CMT network for SIM and CSM. 

## 2. Materials and Methods

### 2.1. Proposed Framework

**Figure 1:**
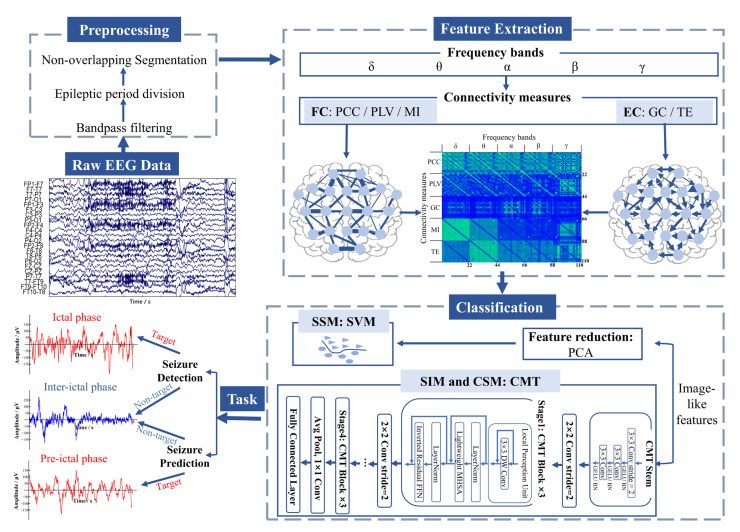
The block diagram of the proposed framework. FC: functional connectivity; EC: effective connectivity; PCC: Pearson correlation coefficient; PLV: phase locking value; MI: mutual information; GC: Granger causality; TE: transfer entropy; PCA: principal component analysis; SSM: subject-specific model; SIM: subject-independent model; CSM: cross-subject model; SVM: Support Vector Machine; CMT: CNNs Meet Transformers; Conv: convolution; BN: batch normalization; DW Conv: depth-wise convolution; MHSA: multi-head self-attention; FFN: feed-forward network; Avg Pool: average pooling.

The workflow is illustrated in Figure 1. First, multichannel EEG raw data were preprocessed, including bandpass filtering, epileptic period division (inter-ictal, pre-ictal, and ictal), and segmentation. Second, five physiological bands were extracted, and five connectivity measures were adopted for each band, including three FC and two EC measures. Third, all connection matrices were combined into a large one as an image-like feature. We chose SVM as the classifier for SSM and CMT for SIM and CSM. Finally, the performance of the model was evaluated. Optimal features, including connectivity measures and frequency bands, were selected, and the efficiency was analyzed in practical applications.

### 2.2. EEG Datasets

**Figure 2:**
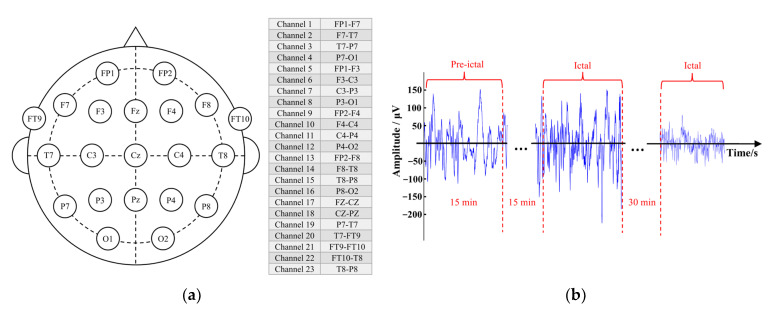
Montage A and periods of CHB-MIT EEG data. (**a**) The electrode placement in Montage A. Most EEG signals were recorded using the international 10–20 electrode system, and two electrodes (FT9 and FT10) were based on the 10–10 electrode system. The EEG channels adopting bipolar montage are on the right, where each electrode’s voltage is linked and compared to an adjacent one to form a chain of electrodes. The bipolar montage can offer better artifact rejection than referential montages, and it is free of volume conduction problems [24,25,26]. (**b**) The definition of different EEG states. The ictal phase was extracted according to experts’ manual marks. 15 to 30 min before the onset of each seizure was defined as the pre-ictal period, so the SPH here was 15–30 min [27]. The inter-ictal state was within an interval between half an hour after the end of a seizure and before the onset of the next pre-ictal state [28].

We adopted the free online dataset CHB-MIT [22], which includes 23 scalp EEG sets collected from 23 people with epilepsy. Details are presented in Table 1. ID 21 and ID 01 are from the same subject with an interval of 1.5 years, but the two recordings were treated as two different cases here due to the long interval [23]. There were different montages in CHB-MIT, including Montage A (23-channel), as shown in Figure 2a, and Montage B (28-channel: Montage A + 5 “virtual” signal channels). Most of the recordings adopted Montage A or its extensions. In Figure 2a, the EEG channels used bipolar reference to estimate the potential differences between two adjacent electrodes. This bipolar montage can offer better artifact rejection and sharper spatial localization than referential montages [24,25]. Moreover, as a non-potential but the derivative of the potential, bipolar EEG is far away from volume conduction problem [24,26]. The sampling rate is 256 Hz. Experts manually marked the start and end times of the seizures.

### 2.3. Preprocessing

All extra channels were removed for all extended montages of A. For example, five “virtual” signal channels out of Montage B were deleted. Channel order was rearranged to keep it the same as Montage A. Channel 23 was deleted because it is identical to channel 15. In this manner, we obtained 22-channel EEG data from 24 cases. In a pre-experiment, we designed two band-pass filters at 0.5–40 Hz and at 0.5–100 Hz to process the EEG recordings and conducted the classification tasks. Results showed that the filter at 0.5–100 Hz contributed more to seizure detection while the filter at 0.5–40 Hz worked better on seizure prediction. More details and discussion are shown in Supplementary Material. Therefore, for seizure detection, a Butterworth filter at 0.5–100 Hz was applied to capture high-frequency features of the ictal phase, and a notch filter at 60 Hz to remove power-line noise. For seizure prediction, a Butterworth filter at 0.5–40 Hz was used to remove high-frequency noise and keep useful information on the pre-ictal phase [29]. 

Next, different EEG states [27,28] were extracted, as shown in Figure 2b. Subsequently, two non-overlapping rectangular windows with different lengths (1 s and 8 s) were applied to segment the EEG signals. It has been reported that long windows have higher classification accuracy, whereas short windows have faster speed of calculating features [6]. Finally, two datasets were prepared, one containing ictal and inter-ictal EEG segments for seizure detection and the other including pre-ictal and inter-ictal phases for seizure prediction. In seizure detection, because the duration of a seizure event was much shorter than that of inter-ictal periods, we made the number of inter-ictal segments consistent with that of the ictal state for sample balance between classes. The number of ictal segments is listed in Table 1. The total 1-s segments of each category was 11,051, and the total 8-s segments was 1321. In seizure prediction, only the pre-ictal and inter-ictal phases were required, which indicated that the number of segments were not greatly limited. Therefore, we prepared for each subject 800 1-s segments and 100 8-s segments of each class, resulting in the total 19,200 1-s segments and 2400 8-s segments of each category.

### 2.4. Feature Extraction

To extract rich features, a bandpass filter was employed to transfer each EEG segment into five physiological frequency bands [30]: δ (0.5–4 Hz), θ (4–8 Hz), α (8–13 Hz), β (13–30 Hz), and γ (30–40 Hz). There are three types of brain connections [12,13]: (1) structural connectivity, an anatomical connection between brain neurons; (2) functional connectivity (FC), a statistical interdependence between different neuronal activities, which belongs to undirected connection; (3) effective connectivity (EC), the causal effect of one neural region on another, which belongs to directed connection. Only FC and EC were calculated in each band because structural connectivity cannot be obtained using EEG data. We selected measures based on different mathematical assumptions to obtain various adjacency matrices of a brain network. 

#### 2.4.1. Functional Connectivity Estimate

1. Pearson Correlation Coefficient (PCC)

First, we assume $x_{p}\left(t\right)\;\left\{p=1,2,\ldots ,22\right\}$ is an EEG signal in the *p*-th channel. As a fast and simple measure, PCC estimates the linear correlation between two signals $x_{p}\left(t\right)$ and $x_{q}\left(t\right)$ in a time domain, ranging from −1 to 1. The formula is shown in Equation (1):

(1) $$\rho_{pq}=\frac{E\left[\left(x_{p}\left(t\right)-\mu_{p}\right)\left(x_{q}\left(t\right)-\mu_{q}\right)\right]}{\sigma_{p}\sigma_{q}}$$

where *E* is the mathematic expectation. $\mu_{p}$ and $\mu_{q}$ are respectively the mean value of $x_{p}\left(t\right)$ and $x_{q}\left(t\right)$. $\sigma_{p}$ and $\sigma_{q}$ are the standard deviation of $x_{p}\left(t\right)$ and $x_{q}\left(t\right)$, separately.

2. Phase Locking Value (PLV)

Based on phase synchronization, PLV [31] measures FC by calculating the synchronization strength instantaneous of phase between two signals. It assumes that two bioelectrical signals with asynchronous amplitudes may be synchronized in phase showing simultaneous (or fix-delayed) rise and fall of two phases. The instantaneous phase of $x_{p}\left(t\right)$ is given by Equation (2):

(2) $${Ø}_{p}\left(t\right)=arctan\frac{{\tilde{x}}_{p}\left(t\right)}{x_{p}\left(t\right)}$$

where ${\tilde{x}}_{p}\left(t\right)$ represents the Hilbert transform of $x_{p}\left(t\right)$, which is defined in Equation (3):

(3) $${\tilde{x}}_{p}\left(t\right)=\frac{1}{\pi }P.V.\int_{-\infty }^{+\infty }\frac{x_{p}\left(\tau \right)}{t-\tau }d\tau$$

where *PV* is the Cauchy principal value. Moreover, the *PLV* value between two signals is calculated according to Equation (4):

(4) $$PLV=\left|\frac{1}{N}\overset{N-1}{\underset{n=0}{\sum }}e^{j\left({Ø}_{p}\left(n∆t\right)-{Ø}_{q}\left(n∆t\right)\right)}\right|$$

where ∆t is the sampling period, and *N* denotes the number of sampling points of signal. The *PLV* values range from 0 to 1.

3. Mutual Information (MI)

Based on information theory, MI evaluates the information dependency between two random variables. It represents the information amount of one signal contained in another. When we use $X_{p}$ = $\left\{x_{p}^{k}|\;x_{p}^{k}=x_{p}\left(t=\left(k-1\right)∆t\right),\;k=1,2,\ldots ,N\right\}$ as the random variable format of $x_{p}\left(t\right)$, the calculation of its entropy $H\left(X_{p}\right)$ is given in Equation (5):

(5) $$H\left(X_{p}\right)=-\overset{N}{\underset{k=1}{\sum }}P\left(x_{p}^{k}\right)log\left(P\left(x_{p}^{k}\right)\right)$$

$H(X_{q}|X_{p})$ and $H\left(X_{p},X_{q}\right)$ indicate the conditional entropy and joint entropy between $X_{p}$ and $X_{q}$, which are defined in Equations (6) and (7):

(6) $$H\left(X_{p},X_{q}\right)=-E_{X_{p}}\left[E_{X_{q}}\left[logP\left(X_{p},X_{q}\right)\right]\right]$$

(7) $$H\left(X_{q}|X_{p}\right)=-E_{X_{p}}\left[E_{X_{q}}\left[logP\left(X_{q}|X_{p}\right)\right]\right]$$

where the MI value between $X_{p}$ and $X_{q}$ is given in Equation (8):

(8) $$MI\left(X_{p},X_{q}\right)=H\left(X_{p}\right)+H\left(X_{q}\right)-H\left(X_{p},X_{q}\right)=H\left(X_{q}\right)-H\left(X_{q}|X_{p}\right)$$

#### 2.4.2. Effective Connectivity Estimate

1. Granger Causality (GC)

GC [32] has an intuitive assumption: if the historical information of $x_{p}\left(t\right)$ can contribute to predicting the future changes of $x_{q}\left(t\right)$, it is considered that $x_{p}\left(t\right)$ is the Granger cause of $x_{q}\left(t\right)$. Firstly, the linear autoregressive (AR) models corresponding to $x_{p}\left(t\right)\;$ and $x_{q}\left(t\right)$ are constructed according to Equations (9) and (10):

(9) $$x_{p}\left(t\right)=\overset{d}{\underset{\tau =1}{\sum }}A_{11\tau }x_{p}\left(t-\tau \right)+\eta_{p}\left(t\right)$$

(10) $$x_{q}\left(t\right)=\overset{d}{\underset{\tau =1}{\sum }}A_{22\tau }x_{q}\left(t-\tau \right)+\eta_{q}\left(t\right)$$

where *d* is the order of the AR models. $A_{11\tau }$ and $A_{22\tau }$ represent the AR coefficient. The mean values of noise $\eta_{p}\left(t\right)$ and $\eta_{q}\left(t\right)$ are both zero and their variances $\sum_{1}=var\left(\eta_{p}\left(t\right)\right)$ and $\sum_{2}=var\left(\eta_{q}\left(t\right)\right)$ are unrelated. The bivariate AR model is defined in Equations (11) and (12):

(11) $$x_{p}\left(t\right)=\overset{d}{\underset{\tau =1}{\sum }}A_{11\tau }x_{p}\left(t-\tau \right)+A_{12\tau }x_{q}\left(t-\tau \right)+e_{p}\left(t\right)$$

(12) $$x_{q}\left(t\right)=\overset{d}{\underset{\tau =1}{\sum }}A_{21\tau }x_{p}\left(t-\tau \right)+A_{22\tau }x_{q}\left(t-\tau \right)+e_{q}\left(t\right)$$

where $A_{12\tau }$ and $A_{21\tau }$ denote the cross-correlation coefficients. The mean values of noise $e_{p}\left(t\right)$ and $e_{q}\left(t\right)$ are both zero, and their variances $\sum_{pp}=var\left(\eta_{p}\left(t\right)\right)$, $\sum_{qq}=var\left(\eta_{q}\left(t\right)\right)$ and covariance $\sum_{pq}=var\left(e_{p}\left(t\right),e_{q}\left(t\right)\right)$ are unrelated. The joint covariance matrix is given in Equation (13):

(13) $$\sum_{noise}=\left[\begin{array}{cc} \sum_{pp} & \sum_{pq}\\ \sum_{qp} & \sum_{qq} \end{array}\right]$$

The overall interdependency between $x_{p}\left(t\right)\;\mathrm{and}\;x_{q}\left(t\right)$ is described in Equation (14):

(14) $$F_{p,q}=ln\frac{\sum_{1}\sum_{2}}{\left|\sum_{noise}\right|}=ln\frac{\sum_{1}}{\sum_{pp}}+ln\frac{\sum_{2}}{\sum_{qq}}+ln\frac{\sum_{pp}\sum_{qq}}{\left|\sum_{noise}\right|}$$

where $\left|\cdot \right|$ denotes the determinant of the enclosed matrix. When the two time series are independent, $F_{p,q}$ reaches 0. $F_{p,q}$ is decomposed into three items: (1) the GC value from $x_{q}\left(t\right)$ to $x_{p}\left(t\right)$; (2) the GC value from $x_{p}\left(t\right)$ to $x_{q}\left(t\right)$; and (3) the instantaneous causality between the two signals. We usually use the first two items to measure the GC values in different directions.

2. Transfer Entropy (TE)

TE has a similar assumption as GC, but it derives from information entropy; if the historical information of the random process $X_{p}$ can help reduce the uncertainty (entropy) of the random process $X_{q}$, it is considered that $X_{p}$ is the cause of $X_{q}$. The calculation of TE value from $X_{p}$ to $X_{q}$ is shown in Equation (15):

(15) $$TE_{p\to q}=H\left(X_{q}^{k}|X_{q}^{k-1:k-d}\right)+H\left(X_{p}^{k}|X_{q}^{k-1:k-d}\right)-H\left(X_{q}^{k},X_{p}^{k-1:k-d}|X_{q}^{k-1:k-d}\right)$$

where *d* is the time lag deciding the time length of historical information. $X_{p}^{k-1:k-d}$ and $X_{q}^{k-1:k-d}$ represent the historical information of $X_{p}$ and $X_{q}$. When the two random processes are independent, the *TE* value equals 0. 

#### 2.4.3. Connectivity Features Arrangement

**Figure 3:**
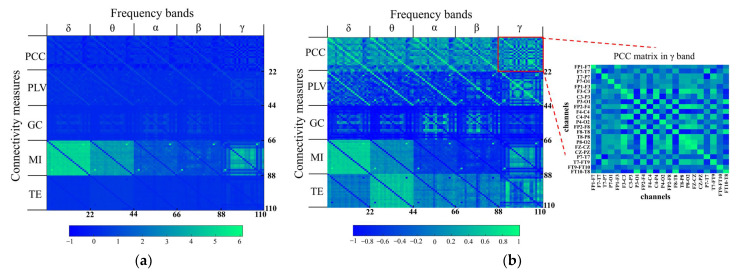
Connectivity feature visualization before and after normalization. The brain connectivity feature image of 110 × 110 comprised 25 connectivity adjacency matrixes (size: 22 × 22). Every 22 columns from left to right represent the features obtained in δ, θ, α, β, and γ, respectively. Every 22 rows from top to bottom represent the features calculated by PCC, PLV, MI, GC, and TE, respectively. (**a**) Brain connectivity image before normalization. The ranges of connectivity values measured by different methods differed, making the difference of most feature elements unobvious. (**b**) Brain connectivity image after normalization. The matrix in the red rectangular box indicated the adjacency matrix computed in γ band using PCC method. All the connectivity values between each pair of channels were arranged in the form of adjacency matrix according to the channel order. The horizontal and vertical axes of this matrix represented the order and names of channels. All the connectivity values corresponding to each measure were normalized to [−1, 1]. The differences among most feature elements were more obvious for classifier learning.

For a 22-channel EEG segment in a specific frequency band *b*, connectivity measure *m* was calculated for each pair of channels, resulting in a 22 × 22 brain connectivity adjacency matrix ${\left\{C_{ij}\right\}}_{b}^{m}$ (channel *i*, *j* ∈ {1, 2, 3, …, 22}; measure m ∈ {PCC, PLV, MI, GC, TE}; band b ∈ {δ, θ, α, β, γ}). For FC measure, the element $c_{ij}$ represented an undirected connectivity between the EEG signals in the *i*-th and the *j*-th channels. For EC method, $c_{ij}$ denoted a directed connectivity from the *i*-th and the *j*-th channels. Finally, 25 (5 bands × 5 measures) adjacency matrices were extracted. Referring to [33], we arranged these matrices to obtain a brain connectivity feature image (110 × 110), as shown in Figure 3a. The differences in most feature elements were not obvious, which could be mitigated by normalizing all the connectivity values corresponding to each *m*, as shown in Figure 3b. After this normalization, the feature diversity was more obvious for classifier learning. The details of the feature dataset were shown in Table 2.

### 2.5. Classification

#### 2.5.1. Classifiers

1. Support Vector Machine (SVM)

SVM [34] was selected as a powerful classifier that effectively performs the nonlinear classification of high-dimensional features. The SVM is designed to find the hyperplane farthest from different sample boundaries. The kernel function plays a very important role in SVM, which can solve nonlinear problems and replace the inner product operation in high-dimensional feature space to avoid the complexity of high-dimensional operations. Common kernel functions include linear, polynomial, and radial basis function (RBF) kernels. Due to its strong nonlinear mapping ability, an RBF was selected here. In addition, SVM has two additional parameters: penalty factor C and kernel parameter *g*. The former represents the tolerance of the error, and the latter implicitly determines the distribution of the data mapped to the new feature space. A grid search determined the optimal parameters of the SVM. The range of the grid search for parameter C was set as [0.001, 0.01, 0.1, 1, 10, 100, 1000], while the search range for parameter *g* was [0.0001, 0.001, 0.01, 0.1, 1, 10, 100]. The decision formula of SVM was given in Equation (16):

(16) $$f\left(x\right)=\overset{n}{\underset{i=1}{\sum }}a_{i}y_{i}K\left(x_{i},x\right)+b$$

where *f*(*x*) is a predicted label, *n* is the number of training samples, *a_i_* represents a Lagrange multiplier, *y_i_* is the label of the *i*-th sample, *x* denotes a feature vector input, *x_i_* indicates the *i*-th sample, *b* means a bias term, and *K*(*x_i_*, *x*) is an RBF kernel, as shown in Equation (17):

(17) $$K\left(x_{i},x\right)=exp\left(-g\times {\left\|x_{i}-x\right\|}^{2}\right)$$

where *g* is a kernel parameter controlling RBF’s radial action range.

2. CNNs Meet Transformers (CMT)

CMT [21] is a hybrid network relying on CNN and transformer to extract local and global information, respectively. The architecture of CMT network is shown in Table S3 in Supplementary Material. It first employs a convolutional stem, which uses multiple 3 × 3 convolution stacks for downsampling and detailed features. Its main body consists of a four-stages transformer. Each stage is formed by stacks of CMT blocks, each of which includes a local perception unit (LPU), a lightweight multi-head self-attention (LMHSA) module, and an inverted residual feed-forward network (IRFFN). The LPU is defined in Equation (18), which introduces a shortcut for stable training:

(18) $$\mathrm{LPU}\left(X\right)=\mathrm{DWConv}\left(X\right)+X$$

where $X\in R^{H\times W\times d}$(*H* × *W* is the resolution of the input of the current stage and d indicates the dimension of features), and DWConv(**·**) denotes the depth-wise convolution. The lightweight attention in the second module is shown in Equation (19):

(19) $$\mathrm{LightweightAttention}\left(Q,\;K,V\right)=\mathrm{Softmax}\left(\frac{{QK}^{\prime }{}^{T}}{\sqrt{d_{k}}}+B\right)V^{\prime }$$

where $Q\in R^{n\times d_{k}}$, $K\in R^{n\times d_{k}}$, and **V**$\in R^{n\times d_{v}}$ represent query, key, and value in the original self-attention module. The notation *n* = *H* × *W* is the number of patches. *d_k_* and *d_v_* denote the query (key) and value dimensions, respectively. $K^{\prime }=\mathrm{DWConv}\left(K\right)\in R^{n/k^{2}\times d_{k}}$ and $V^{\prime }=\mathrm{DWConv}\left(V\right)\in R^{n/k^{2}\times d_{v}}$ are obtained for lightweight features. is randomly initialized and learnable. Finally, *h* LighweightAttention functions are the *h* “heads” of LMHSA, resulting in a final *n* × *d* sequence. Compared with an FFN in traditional transformers, the IRFFN given in Equation (20) applies a depth-wise convolution to extract local information with negligible computational cost.

(20) $$\mathrm{IRFFN}\left(X\right)=\mathrm{Conv}\left(F\left(\mathrm{Conv}\left(X\right)\right)\right)$$

where Conv (**·**) denotes a traditional convolution and $F\left(X\right)=\mathrm{DWConv}\left(X\right)+X$. Therefore, the information is passed through a CMT block, as shown in Equations (21)–(23):

(21) $$Y_{i}=\mathrm{LPU}\left(X_{i-1}\right)$$

(22) $$Z_{i}=\mathrm{LMHSA}\left(LN\left(Y_{i}\right)\right)+Y_{i}$$

(23) $$X_{i}=\mathrm{IRFFN}\left(LN\left(Z_{i}\right)\right)+Z_{i}$$

where **Y***_i_*, **Z***_i_*, and **X***_i_* denote the output of LPU, LMHSA, and IRFFN in the *i*-th block, respectively. LN (**·**) represents layer normalization. Finally, CMT ends with a global average pooling layer, a projection layer, and a classification layer with softmax. According to the experimental experience, sigmoid rather than softmax was used here. We selected cross-entropy loss and L2-norm as the loss function and the regularization term, respectively.

#### 2.5.2. Performance Evaluation Metrics

This study adopted three widely used evaluation criteria for seizure detection and prediction: accuracy (*ACC*), sensitivity (*Sen*), and specificity (*Spe*). *ACC* represents the percentage of correct period detection, *Spe* represents the percentage of correct inter-ictal EEG recognition, and *Sen* represents the percentage of correct interest period identification. For seizure detection, the ictal phase is the interest period, whereas, for seizure prediction, the pre-ictal period is the interest period. The calculation formulas for performance evaluation criteria are shown in Equations (24)–(26):

(24) $$ACC=\frac{TP+TN}{TP+FN+TN+FP}\times 100\%$$

(25) $$Sen=\frac{TP}{TP+FN}\times 100\%$$

(26) $$Spe=\frac{TN}{TN+FP}\times 100\%$$

where *TP* denotes the number of samples correctly identified as the interest period, *FN* represents the number of samples incorrectly identified as the inter-ictal segment, *TN* refers to the number of samples correctly identified as the inter-ictal period, and *FP* is the number of samples incorrectly identified as the interest period.

#### 2.5.3. Training and Validation Strategy

1. Subject-Specific Model (SSM)

**Figure 4:**
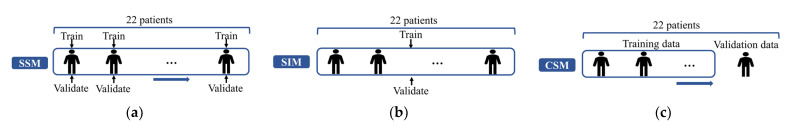
Training and validation strategies for (**a**) SSM, (**b**) SIM, and (**c**) CSM.

Figure 4a showed the SSM training and verification processes. Since the training and validation data for the SSM were both from the same individual, the obtained samples were limited. With the advantage of small sample classification, SVM was adopted to build the SSM. However, as the SVM cannot directly process image input, the brain connectivity matrix (110 × 110) was flattened into a 12,100-dimensional feature vector whose dimensions were too high to suit the SVM. Therefore, principal component analysis (PCA) [35] was used to extract low-dimensional main components as the feature vector input of the SVM. Based on practical experience, we selected the first 100 principal components of the 1 s-features and the first 30 principal components of the 8 s-features as the input features. A five-fold cross-validation was used to provide an unbiased evaluation of its performance and avoid overfitting. Then the results were averaged across folds to verify a single model. Finally, these evaluations were averaged across subjects to check the average performance of the SSM.

2. Subject-Independent Model (SIM)

Figure 4b outlined how SIM was trained and verified. First, the EEG segments of all the subjects were collected to form a dataset. The ratio of the training data and validation data was set to 8:2. In the training process, we set the initial learning rate to 0.0001 (decayed every five epochs), the regularization coefficient to 0.001, and batch size to 8. Finally, the trained CMT used the validation set to verify the performance of the SIM.

3. Cross-Subject Model (CSM)

Figure 4c showed the CSM’s leave-one-subject-out (LOSO) validation. One subject was left as the validation data, and the remaining subjects formed training data. CMT was used to establish CSM. The evaluations were averaged across subjects to check the average performance of the CSM. We set the learning rate to 0.0001 (decayed every five epochs), the regularization coefficient to 0.01, and the batch size to 8. 

#### 2.5.4. Feature Selection and Efficiency Analysis

**Figure 5:**
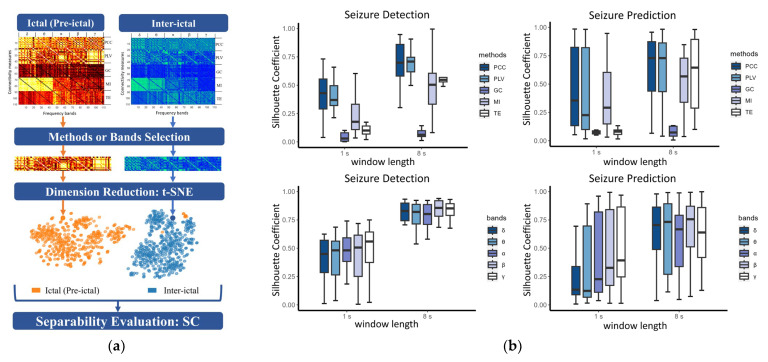
Feature selection (**a**) strategy and (**b**) results. The results include the comparison of SC among connectivity methods for seizure detection (top-left) and prediction (top-right) and the comparison of SC among frequency bands for seizure detection (bottom-left) and prediction (bottom-right). The SC corresponding to each method was calculated based on the features in all five frequency bands. The SC corresponding to each band was calculated based on the features extracted by PCC and PLV methods.

To determine the optimal connectivity measures and frequency bands, we evaluated the feature separability between different epileptic phases, as shown in Figure 5a. First, the features corresponding to a specific method or band were extracted. Since the distance of feature vectors in high-dimensional space is difficult to measure accurately, t-distributed stochastic neighbor embedding (t-SNE) [36] was adopted to map a high-dimensional feature to a point in a two-dimensional plane. It uses a heavy-tailed distribution (like the Student t-distribution) to convert distances into probability scores in low dimensions, which results in points with greater similarity in high-dimensional space being mapped to points with smaller distances in low-dimensional space and vice versa. Next, according to Equation (27), a silhouette coefficient (*SC*) was calculated for clusters formed by two classes of feature points on the plane, positively related to the degree of feature separation.

(27) $$SC=\frac{1}{N}\overset{N}{\underset{i=1}{\sum }}sc\left(i\right)$$

where *N* is the number of samples, and *sc*(*i*) is the silhouette coefficient of the *i*-th sample, given in Equation (28).

(28) $$sc\left(i\right)=\frac{b\left(i\right)-a\left(i\right)}{max\left\{a\left(i\right),b\left(i\right)\right\}}$$

where *b*(*i*) and *a*(*i*) represent the average distance between the *i*-th sample and other samples with a different and the same label, respectively. The *SC* corresponding to each method was calculated for each subject. A Shapiro-Wilk test showed the failure of the *SC* values to conform to a (nearly) normal distribution, and a Spearman Correlation test detected the significant correlations (*p* < 0.01) among the five methods. Therefore, a non-parametric multiple-paired test, namely, the Friedman test was conducted among the *SC* values of the five methods, and the *p*-values were corrected by Finner correction. 

After selecting optimal connectivity measures, the SC was computed for each frequency band and the optimal measures. Due to the non-normal distribution and the significant correlations among the five bands, the same statistical method was applied for optimal band selection. After the above feature selection, we analyzed the efficiency of optimal features. In general, a practical method in real application scenarios must have high efficiency in terms of time and storage to achieve online real-time detection and prediction. Therefore, the time and storage costs were calculated as efficiency metrics. All computations were performed on a standard desktop computer with an Intel Core i7-10700 CPU @ 2.90 GHz processor and 16 GB RAM. Given that the training time of the model largely depends on the training sample size, which was not considered in this study, we focused on the time spent on preprocessing, feature extraction, and model output. The storage cost was local-specific. 

## 3. Results

### 3.1. Classification Results

Table 3 presented the classification results for the proposed method. All evaluation criteria for SSM and SIM were greater than 95%. In the 8 s window, ACC, Sen, and Spe of SSM and SIM reached more than 99%. For seizure detection, the CSM obtained satisfactory results, with each metric over 96%. However, the performance of CSM decreased significantly in seizure prediction. In the 1 s- and 8 s-windows, the seizure prediction ACC reached 74.78% and 86.17%, respectively. 

### 3.2. Feature Selection

The statistical results were displayed in Figure 5b. For seizure detection, PCC and PLV had significantly higher SC than GC (both windows: *p* < 0.001), MI (1 s window: *p* < 0.01; 8 s window: *p* < 0.05), and TE (1 s window: *p* < 0.001; 8 s window: *p* < 0.01). MI showed better feature separability than GC (both windows: *p* < 0.01). For seizure prediction, PCC and PLV had larger SC than GC (both windows: *p* < 0.001), MI (8 s window: *p* < 0.05), and TE (1 s window: *p* < 0.001). Lower SC was obtained for GC than MI (1 s windows: *p* < 0.001) and TE (8 s window: *p* < 0.001). Based on the results above, the features calculated by PCC and PLV showed optimal separability. Therefore, we extracted the PCC and PLV connection matrix (44 × 22) in each frequency band for SC comparison. For seizure detection in the 1 s window, γ band had higher SC than δ (*p* = 0.001), θ (*p* = 0.001), and α (*p* = 0.030), and β showed better feature separability than δ (*p* = 0.003) and θ (*p* = 0.004). For seizure detection in the 8 s window, β band produced higher SC values than δ (*p* = 0.039), θ (*p* = 0.028), and α (*p* = 0.028). γ band showed better feature separability than θ (*p* = 0.028) and α (*p* = 0.039). For seizure prediction in the 1 s window, β and γ had significantly higher SC than δ (*p* < 0.001), θ (*p* < 0.001), and α (*p* < 0.05) bands. There was no difference among the frequency bands for seizure prediction in the 8 s window.

Based on the results above, we recommend a feature selection scheme: PCC and PLV connectivity features in the β- and γ-bands. The classification ACC, time cost, and storage cost comparisons between the original and selected features were shown in Table 4. After removing the other three measures and three bands, this selection scheme’s time and storage costs substantially decreased by more than 70%, but the ACC only decreased by less than 3.50%. 

## 4. Discussion

### 4.1. Classification Results

#### 4.1.1. Training and Validation Strategy Comparison

In the 1 s window, there were obvious differences in the performance of the different training and validation strategies. From the perspective of data distribution, the EEG data pattern distribution on the same individual should be relatively similar while the distributions among different individuals have significant diversity, which was named cross-subject heterogeneity [37]. In this case, SSM should have the best classification performance than SIM and CSM. However, in this study, SSM and SIM reached similar accuracy. For one, the training and validation sets of SIM are likely to come from the same subjects, which mitigated the cross-subject diversity to some extent. For another, CMT was used for SIM construction, with a stronger fitting ability than SVM-based SSM. Nonetheless, CMT’s powerful ability failed to overcome the pronounced heterogeneity problem in CSM for seizure prediction. Although SIM could work on different subjects and have higher accuracy than CSM, it has limitations in real application scenarios. Since SIM was trained and validated on the same subject group, it could not work well on a “never-seen” subject. If it is to work on the new subject, it is necessary to collect her/his EEG data and add it to the dataset for model training. The ideal situation is that a model can still work on brand-new subjects without training. Therefore, the CSM has the highest practical value among the three models. Recently, Giuseppe et al. [38] have attempted LOSO validation in the classification between EEG signals of psychogenic nonepileptic seizures (PNES) and of the healthy subjects, obtaining a good accuracy. However, most studies in seizure detection and prediction did not adopt LOSO validation; therefore, we suggest that CSM’s training and verification strategy be used in this field to provide a model evaluation in real application scenarios.

#### 4.1.2. Window Length Comparison

The long window performed better than the short window, which is consistent with the literature [6]. A possible reason is that brain connectivity values may become noisier in a short segment due to reduced sample data points [39]. Another reasonable explanation might be that some connectivity measures require additional samples to detect a phase shift in a particular band. Therefore, features extracted from longer segments are more likely to be stable to carry more useful information. However, there are some limitations to its practical application. Without knowing the period labels, a randomly intercepted 8 s segment has a high chance of including two different periods simultaneously (e.g., inter-ictal and pre-ictal states), which greatly affects the classification accuracy. In addition, a long window mitigates the feature predominance of some seizures with a short duration, leading to missed detection. Therefore, we suggest selecting an appropriate window length according to specific requirements.

#### 4.1.3. Comparison with Previous Studies

A comparison of the seizure detection results was displayed in Table 5. Our classification performances of SSM and SIM in the 1 s window were better than the previous results based on the short window (1~2 s). When the window length was 8 s, our SSM, SIM, and CSM results reached satisfactory values of over 99%, higher than other studies. These comparisons showed that the brain connectivity features were effective in seizure detection. Similarly, brain connectivity has been recently proven as a promising feature for the classification between rest-EEG data of PNES from healthy control subjects [40].

A comparison of the results for seizure prediction was presented in Table 6. The difficulty of seizure prediction is sensitive to SPH, which makes SPH a key factor for comparison. In the 1 s and 8 s window, our results reached the state-of-the-art as every evaluation criterion of SSM and SIM was over 96%, which was higher than other results. In addition, CMT-based SIM reached an accuracy of over 96% in both window lengths, which indicated that CMT succeeded in useful features on brain network learning. However, CMT-based CSM did not reach satisfactory performance. Careful examination reported that the ACC varied significantly across individuals, ranging from 60% to 100%, which resulted from the large heterogeneity of individual EEG data. 

### 4.2. Feature Selection

#### 4.2.1. Connectivity Measure Comparison

The results in Section 3.2. indicated that the two EC methods performed worse than the three FC measures. Since related studies have shown that there were obvious differences between effective connection networks in different EEG states [14,15], we believe that the main reason EC was suboptimal here was that the selected GC and TE methods could not accurately measure the true effective connectivity of EEG data from the CHB-MIT dataset. Although GC is a widely used measure of causality, it has an established shortcoming of high sensitivity to noise, which becomes particularly acute with noisy electrophysiological recordings such as scalp EEG signals [48]. The increase in noise was likely to cause a change in the GC direction and the appearance of false connections. Furthermore, if there is a third unmeasured random process that affects the two signals simultaneously, the measurement accuracy of TE will be severely affected [49]. Therefore, we do not recommend traditional GC and TE methods to extract effective connectivity for seizure detection and prediction. Although MI showed relatively good separability, it required a long computational time. In contrast, PCC and PLV are recommended candidates with good effect, easy operation, and a fast computing speed. They provide connectivity information in a brain network from the time and frequency domains.

#### 4.2.2. Frequency Band Comparison

The results in Section 3.2. indicated that features calculated in the β and γ bands were more effective, which is consistent with the following evidence. From a pathological perspective, observing human and animal epilepsy models showed a relationship between the epileptogenicity of neuronal tissue and its tendency to produce rapid oscillations during seizures. The frequency of seizures gradually increases during the transition from the pre-ictal to the ictal state [50]. From clinical observations, removing brain areas with rapid discharge could positively impact the prognosis of surgery [51]. Blanco et al. [52] analyzed the Fourier spectral entropy of EEG signals within the pre-ictal period based on the Freiburg dataset. They found that the high-frequency spectral entropy in the pre-ictal period significantly increased compared with that in the inter-ictal phase, which indicated that the abnormal information transmission generated during the discharge of neuronal clusters in the focus area might result in signal energy transfer from the low-frequency to the high-frequency band. Other researchers have also used experiments to prove that β and γ oscillations could be biomarkers of seizures [53,54,55]. Therefore, we suggest β and γ bands as effective frequency bands for seizure detection and prediction. 

### 4.3. Limitations

There were three limitations: (1) Although bipolar montage is far away from volume conduction, this problem is too pronounced to be solved comprehensively. Moreover, when EEG recordings adopt referential (or unipolar) montage, some brain connectivity measures such as PLV should be cautiously treated because they are sensitive to volume conduction. (2) The cross-subject model in seizure prediction failed to perform satisfactorily, indicating that the heterogeneity was not well mitigated. In the next work, other network architectures, such as graph neural networks, will be conducted to solve this problem. (3) The small sample size of CHB-MIT might affect the performance of deep learning networks. Therefore, more clinical data will be collected in our cooperative hospital for classification in the future.

## 5. Conclusions

The main contributions of this work included the following:

(1) the proposed functional and effective connectivity features for seizure detection and prediction: PCC, PLV, GC, MI, and TE;
(2) comprehensive evaluation of the effectiveness of brain connectivity features under different classification tasks (seizure detection and prediction), window lengths (1 s and 8 s), and model training and verification strategies (i.e., SSM, SIM, and CSM);
(3) the classifiers applicable to different training and validation strategies: SVM for SSM, and CMT for SIM and CSM;
(4) the optimal selection of frequency bands and connectivity measures: PCC and PLV features in the β and γ bands;
(5) the classification accuracy and the time-storage efficiency analysis showed the practicality in clinical applications.

## Figures and Tables

**Table 1:** Information about the selected records of the CHB-MIT database.

Patient ID	Gender ^1^	Age (Year)	Number of Channels	Number of Seizures ^2^	Number of 1-s Segments	Number of 8-s Segments
1	Female	11	23	7	442	53
2	Male	11	23	3	172	21
3	Female	14	23	7	402	47
4	Male	22	23	4	378	45
5	Female	7	23	5	558	68
6	Female	1.5	23	10	153	14
7	Female	14.5	23	3	325	39
8	Male	3.5	23	5	919	113
9	Female	10	23	4	276	32
10	Male	3	23	7	447	53
11	Female	12	28	3	806	100
12	Female	2	28	27	989	112
13	Female	3	28	10	440	51
14	Female	9	28	8	169	20
15	Male	16	38	20	2012	252
16	Female	7	28	8	69	8
17	Female	12	28	3	293	36
18	Female	18	28	6	317	36
19	Female	19	28	3	236	28
20	Female	6	28	8	294	32
21	Female	13	28	4	199	24
22	Female	9	28	3	204	25
23	Female	6	28	7	424	49
24	Unknown	Unknown	23	15	527	63
sum				180	11,051	1321

^1^ The evident gender bias (male/female—5/18) exists in CHB-MIT dataset. ^2^ The number of seizures was counted only for Montage A and its extension.

**Table 2:** The details of the feature dataset.

Task	Band-Pass Filtering	Number of Subjects	Number of Samples in Each Class		Feature Size
			1-s Samples	8-s Samples	
detection	0.5–100 Hz	24	11,051 (=$\sum_{p=1}^{24}n_{p}^{1s}$) ^1^	1321 (=$\sum_{p=1}^{24}n_{p}^{8s}$) ^2^	110 × 110
prediction	0.5–40 Hz	24	19,200 (=24 × 800)	2400 (=24 × 100)	110 × 110

^1^ $n_{p}^{1s}$ indicates the number of 1-s ictal segments corresponding to the *p*-th subject (See Table 1). ^2^ $n_{p}^{8s}$ indicates the number of 8-s ictal segments corresponding to the *p*-th subject (See Table 1).

**Table 3:** Classification results based on the proposed method.

Task	Window Length(s)	SSM			SIM			CSM		
		ACC (%)	Sen (%)	Spe (%)	ACC (%)	Sen (%)	Spe (%)	ACC (%)	Sen (%)	Spe (%)
detection	1	100	100	100	99.87	99.75	99.89	96.67	97.00	96.34
	8	100	100	100	99.98	100	99.96	99.27	99.51	99.03
prediction	1	96.67	96.34	97.01	97.64	97.65	97.63	74.78	74.14	75.42
	8	99.72	99.66	99.77	99.38	99.75	99.01	86.17	84.98	87.36

**Table 4:** Comparison of ACC and efficiency between original and selected features.

Task	Window Length (s)	Model	ACC			Time Cost Per 1 h EEG ^4^			Storage Cost for Locality ^5^		
			Original ^1^ (%)	Selection ^2^ (%)	Decline ^3^ (%)	Original (s)	Selection (s)	Decline (%)	Original (KB)	Selection (KB)	Decline (%)
detection	1	SSM	100	99.65	0.35	6158.41	79.61	98.71	79.50	22.00	72.33
		SIM	99.87	99.43	0.44	6701.04	763.56	88.61	62.50	10.00	84.00
		CSM	96.67	94.89	1.84	6701.04	763.56	88.61	62.50	10.00	84.00
	8	SSM	100	99.72	0.28	7336.63	19.76	99.73	79.50	22.00	72.33
		SIM	99.98	99.83	0.15	7505.78	123.26	98.36	62.50	10.00	84.00
		CSM	99.27	97.67	1.61	7505.78	123.26	98.36	62.50	10.00	84.00
prediction	1	SSM	96.67	95.11	1.61	6158.41	79.61	98.71	79.50	22.00	72.33
		SIM	97.64	95.76	1.93	6701.04	763.56	88.61	62.50	10.00	84.00
		CSM	74.78	72.30	3.32	6701.04	763.56	88.61	62.50	10.00	84.00
	8	SSM	99.72	98.67	1.05	7336.63	19.76	99.73	79.50	22.00	72.33
		SIM	99.38	99.15	0.23	7505.78	123.26	98.36	62.50	10.00	84.00
		CSM	86.17	83.77	2.79	7505.78	123.26	98.36	62.50	10.00	84.00

^1^ Original features referred to PCC, PLV, GC, MI, and TE values calculated in δ, θ, α, β, and γ bands. ^2^ Selected features referred to PCC and PLV values obtained in β and γ bands. ^3^ Decline rate denoted the percentage of decline in the indicator based on the selected features compared to the original features. ^4^ Time cost included the time spent on preprocessing raw EEG, feature extraction, and model output. ^5^ The storage cost was calculated for the local side. The original and selected features occupied 62.5 KB and 10 KB. With only 17 KB, SVM-based SSM could be embedded in the local side, which was required to store both feature matrix and model. However, it was unsuitable for SIM or CSM based on CMT with 101 MB to be loaded locally. Given that they could apply to different subjects, SIM and CSM could be uploaded to the cloud side for online computing. The local side was responsible to extract features and then upload a feature matrix. Therefore, the storage cost of SIM or CSM for locality included only a feature matrix.

**Table 5:** Comparison of classification results in seizure detection.

Researchers	Year	Window Length (s)	SSM			SIM			CSM		
			ACC (%)	Sen (%)	Spe (%)	ACC (%)	Sen (%)	Spe (%)	ACC (%)	Sen (%)	Spe (%)
Zabihi [41]	2016	1	93.11	88.27	93.21	–	–	–	–	–	–
Fergus [42]	2016	60	–	–	–	–	84	85	–	–	–
Zhou [43]	2018	1	97.50	96.90	98.10	–	–	–	–	–	–
Alickovic [27]	2018	8	–	–	–	100	100	100	–	–	–
Raghu [44]	2019	1	–	–	–	–	94.21	–	–	–	–
Selvakumari [45]	2019	1	95.63	95.70	96.55	–	–	–	–	–	–
Wei [23]	2019	5	–	–	–	–	–	–	84.00	72.11	95.89
Akbarian [14]	2020	1.2	99.43	98.67	99.02	–	–	–	–	–	–
Zarei [30]	2021	2	97.09	96.81	97.26	–	–	–	–	–	–
Ours	–	1	100	100	100	99.87	99.75	99.89	96.67	97.00	96.34
Ours	–	8	100	100	100	99.98	100	99.96	99.27	99.51	99.03

**Table 6:** Comparison of classification results in seizure prediction.

Researchers	Year	Window Length (s)	SPH (min)	SSM			SIM		CSM			
				ACC (%)	Sen (%)	Spe (%)	ACC (%)	Sen (%)	Spe (%)	ACC (%)	Sen (%)	Spe (%)
Usman [46]	2017	1	20–33.46	–	92.23	93.38	–	–	–	–	–	–
Zhou [43]	2018	1	–	95.60	94.20	96.90	–	–	–	–	–	–
Zhang [15]	2020	8	0–22.6	89.20	–	–	–	–	–	–	–	–
Jana [6]	2021	1	0–10	93.10	–	–	–	–	–	–	–	–
Jana [6]	2021	2	0–10	96.24	–	–	–	–	–	–	–	–
Jana [6]	2021	8	0–10	99.63	–	–	–	–	–	–	–	–
Zhang [16]	2021	8	0–15	–	–	–	89.98	92.91	87.04	–	–	–
Emara [47]	2021	10	0–30	90.90	–	–	–	83.80	–	–	–	–
Ma [1]	2021	10	0–30	–	–	–	91.40	96.20	19.50	–	–	–
Ours	–	1	15–30	96.67	96.34	97.01	97.64	97.65	97.63	74.78	74.14	75.42
Ours	–	8	15–30	99.72	99.66	99.77	99.38	99.75	99.01	86.17	84.98	87.36

## Data Availability

The data used in this study are available in open access on Physionet. The download link is https://physionet.org/content/chbmit/1.0.0/, accessed on 10 July 2022.

## Supplementary Materials

The following supporting information can be downloaded at: https://www.mdpi.com/article/10.3390/brainsci13050820/s1, Table S1. Classification results with filtering at 0.5–40 Hz; Table S2. Classification results with filtering at 0.5–100 Hz; Table S3. The architecture of CMT network. The feature image was resized to the input resolution of 160 × 160. The output size corresponds to the input resolution. Convolutions and CMT blocks are shown in brackets with the number of stacked blocks. Hi and ki are the number of heads and reduction rates in LMHSA of stage i, respectively. Ri denotes the expansion ratio in IRFFN of stage i. References [21,52] are cited in the supplementary materials.
