# Supplementary material for: Automatic Seizure Detection and Prediction Based on Brain Connectivity Features and a CNNs Meet Transformers Classifier
Source: Brain Sci. 2023 May 18;13(5):820. doi: 10.3390/brainsci13050820 (PMC10216314; doi:10.3390/brainsci13050820)
Supplement: Supplementary file 1 [file brainsci-13-00820-s001.zip › brainsci-2364012-supplementary.pdf]

Supplementary material

In the pre-experiment, two band-pass filters were applied to the EEG recordings for seizure detection and prediction tasks. One had a frequency range from 0.5 to 40 Hz while the other had a range from 0.5 to 100 Hz. Then, the classification results based on the proposed method was described in Table S1 and Table S2.

Table S1. Classification results with filtering at 0.5-40 Hz.

Task	Window length (s)	SSM			SIM			CSM		
		ACC (%)	Sen (%)	Spe (%)	ACC (%)	Sen (%)	Spe (%)	ACC (%)	Sen (%)	Spe (%)
detection	1	98.07	98.02	98.13	94.51	94.06	94.98	81.34	82.01	80.70
	8	99.29	99.29	99.33	99.07	98.99	99.14	95.76	96.56	94.95
prediction	1	94.06	94.04	94.06	96.64	97.08	96.20	72.53	73.15	71.89
	8	98.99	98.99	99.07	99.76	99.81	99.72	85.98	86.54	85.41

Table S2. Classification results with filtering at 0.5-100 Hz.

Task	Window length (s)	SSM			SIM			CSM		
		ACC (%)	Sen (%)	Spe (%)	ACC (%)	Sen (%)	Spe (%)	ACC (%)	Sen (%)	Spe (%)
detection	1	100	100	100	99.87	99.75	99.89	96.67	97.00	96.34
	8	100	100	100	99.98	100	99.96	99.27	99.51	99.03
prediction	1	90.67	88.56	92.78	68.65	70.04	67.26	62.68	63.80	61.56
	8	93.58	93.01	94.15	76.43	77.64	75.22	67.61	67.95	67.27

Table S1 describes the results with a filtering range of 0.5-40 Hz. Table S2 describes the results with a filtering range of 0.5-100 Hz. Comparing the two results, we found an interesting phenomenon: the bandwidth expansion contributed a lot to seizure detection task but had a counterproductive effect on seizure prediction task. Given that seizure is a rapid abnormal discharge process, we speculated that the information included in the high frequency band (> 40 Hz) might be very useful to mark the ictal phase, which could effectively distinguish between ictal and inter-ictal phases.

For pre-ictal EEG signal, the pre-ictal period was defined as 15-30 minutes prior to seizure onset. Blanco, et al. [52] reported that the signal energy transferred from the low-frequency to the high-frequency band in pre-ictal phase. They stated that the greater the entropy was, the more energy flowed into this band. After checking their results carefully, we found that during 15-30 minutes prior to seizures, the overall rate of entropy increase was very slow. It wasn't until 5 minutes before the onset that it began to rapidly climb. This indicated that there has not yet been a significant transfer of signal energy from low to high frequency during 15 and 30 minutes before the onset. That was to say, a large amount of valid EEG information was very likely to be still contained in low and mid frequency bands. Therefore, the gamma band extension from 30-40 Hz to 30-100 Hz might not capture more useful information marking pre-ictal EEG signals but introduce more high-frequency noise, which exactly explained the counterproductive effect of the gamma extension on the seizure prediction.

Based on the above discussion, we finally decided to apply a band-pass filter of 0.5-100 Hz on seizure detection task, and a band-pass filter of 0.5-40 Hz on seizure prediction task. Although gamma band is limited, ranging from 30 only to 40 Hz, we believed that the useful information could be still extracted to mark pre-ictal signals and high-frequency noise would be removed.

Table S3. The architecture of CMT network. The feature image was resized to the input resolution of 160 × 160. The output size corresponds to the input resolution. Convolutions and CMT blocks are shown in brackets with the number of stacked blocks. H_i and k_i are the number of heads and reduction rates in LMHSA of stage i, respectively. R_i denotes the expansion ratio in IRFFN of stage i.

Output Size	Layer Name	CMT[21]
80 × 80	Stem	3 × 3, 32, stride 2<br>[3 × 3, 32] × 2
40 × 40	Patch Embedding	2 × 2, 64, stride 2
Stage 1	LPU<br>LMHSA<br>IRFFN	$\begin{bmatrix} 3 \times 3, 64 \\ H_1 = 1, k_1 = 8 \\ R_1 = 4 \end{bmatrix} \times 3$
20 × 20	Patch Embedding	2 × 2, 128, stride 2
Stage 2	LPU<br>LMHSA<br>IRFFN	$\begin{bmatrix} 3 \times 3, 128 \\ H_2 = 2, k_2 = 4 \\ R_2 = 4 \end{bmatrix} \times 3$
10 × 10	Patch Embedding	2 × 2, 256, stride 2
Stage 3	LPU<br>LMHSA<br>IRFFN	$\begin{bmatrix} 3 \times 3, 256 \\ H_3 = 4, k_3 = 2 \\ R_3 = 4 \end{bmatrix} \times 16$
5 × 5	Patch Embedding	2 × 2, 512, stride 2
Stage 4	LPU<br>LMHSA<br>IRFFN	$\begin{bmatrix} 3 \times 3, 512 \\ H_4 = 8, k_4 = 1 \\ R_4 = 4 \end{bmatrix} \times 3$
1 × 1	Projection	1 × 1, 1280
1 × 1	Classifier	Fully Connected Layer, 1000
